# Identification of functionally important miRNA targeted genes associated with child obesity trait in genome-wide association studies

**DOI:** 10.1186/s12864-022-08576-8

**Published:** 2022-05-11

**Authors:** Melinda Song, Jiaqi Yu, Binze Li, Julian Dong, Jeslyn Gao, Lulu Shang, Xiang Zhou, Yongsheng Bai

**Affiliations:** 1 University of Michigan Medical School, Ann Arbor, MI 48109 USA; 2 College Preparatory School, 6100 Broadway, Oakland, CA 94618 USA; 3 Bellaire High School, 5100 Maple St, Bellaire, TX 77401 USA; 4 Department of Statistics, University of California, Los Angeles, Los Angeles, CA 90095 USA; 5 Northville High School, 45700 Six Mile Road, Northville, MI 48168 USA; 6 College of Engineering, University of Michigan, Ann Arbor, MI 48109 USA; 7 Simsbury High School, 34 Farms Village Rd, Simsbury, CT 06070 USA; 8 Department of Biostatistics, University of Michigan, Ann Arbor, MI 48109 USA; 9 Center for Statistical Genetics, University of Michigan, Ann Arbor, MI 48109 USA; 10 Department of Biology, Eastern Michigan University, Ypsilanti, MI 48197 USA; 11 Next-Gen Intelligent Science Training, Ann Arbor, MI 48105 USA

**Keywords:** GWAS, Variants, Child Obesity, 3’UTR, Functional Enrichment Analysis

## Abstract

**Background:**

Genome-wide association studies (GWAS) have uncovered thousands of genetic variants that are associated with complex human traits and diseases. miRNAs are single-stranded non-coding RNAs. In particular, genetic variants located in the 3’UTR region of mRNAs may play an important role in gene regulation through their interaction with miRNAs. Existing studies have not been thoroughly conducted to elucidate 3’UTR variants discovered through GWAS. The goal of this study is to analyze patterns of GWAS functional variants located in 3’UTRs about their relevance in the network between hosting genes and targeting miRNAs, and elucidate the association between the genes harboring these variants and genetic traits.

**Methods:**

We employed MIGWAS, ANNOVAR, MEME, and DAVID software packages to annotate the variants obtained from GWAS for 31 traits and elucidate the association between their harboring genes and their related traits. We identified variants that occurred in the motif regions that may be functionally important in affecting miRNA binding. We also conducted pathway analysis and functional annotation on miRNA targeted genes harboring 3’UTR variants for a trait with the highest percentage of 3’UTR variants occurring.

**Results:**

The Child Obesity trait has the highest percentage of 3’UTR variants (75%). Of the 16 genes related to the Child Obesity trait, 5 genes (*ETV7, GMEB1, NFIX, ZNF566, ZBTB40*) had a significant association with the term DNA-Binding (*p* < 0.05). EQTL analysis revealed 2 relevant tissues and 10 targeted genes associated with the Child Obesity trait.

In addition, Red Blood Cells (RBC), Hemoglobin (HB), and Package Cell Volume (PCV) have overlapping variants. In particular, the *PIM1* variant occurred inside the HB Motif region 37,174,641–37,174,660, and *LUC7L3* variant occurred inside RBC Motif region 50,753,918–50,753,937.

**Conclusion:**

Variants located in 3’UTR can alter the binding affinity of miRNA and impact gene regulation, thus warranting further annotation and analysis. We have developed a bioinformatics bash pipeline to automatically annotate variants, determine the number of variants in different categories for each given trait, and check common variants across different traits. This is a valuable tool to annotate a large number of GWAS result files.

**Supplementary Information:**

The online version contains supplementary material available at 10.1186/s12864-022-08576-8.

## Background

A microRNA (miRNA) is a single-stranded, non-coding RNA that regulates gene expression. miRNAs can interact with the 3’ untranslated regions (3’UTR) of the targeting messenger RNA (mRNA) and repress protein production by inhibiting translation, a process where genetic information from DNA is transported to the ribosome for protein synthesis [1]. Because of the ability of miRNA to regulate gene expression by controlling development and differentiation, mutations affecting miRNA function could be detrimental. It is widely suggested that mutated miRNAs play a crucial role in human diseases [2].

Genetic variants, such as single nucleotide polymorphisms (SNPs), contribute to diversity among individuals by affecting gene expression. SNPs within the 3’UTR of target mRNAs can influence gene regulation by changing the binding affinity of miRNAs, thus SNPs and their target sites play a significant role in the development of complex traits and diseases [3]. Today, many functional annotation tools and sequencing methods enable convenient and deeper analysis of genetic variation. For example, genome-wide annotation of variants (GWAVA) supports prioritization of noncoding variants and helps predict the functional impact of non-coding variants [4]. Different pathway analysis tools accelerate generation of sequencing data and facilitate identification of functionally relevant variants [4, 5].

Genome-wide association studies (GWAS) uncover thousands of genetic variations associated with diverse human traits and diseases. However, it is difficult to annotate disease influence of non-coding variants (e.g., 3'UTR variants) reported from GWAS studies and explain their impact on binding miRNAs. Recent significant studies that identified GWAS SNPs alter binding sequences have been published. The SMDB database stands out by identifying both losses and gains of important somatic motifs [6]; The updated SomamiR 2.0 database includes somatic mutations for miRNA target recognition [7]. We applied MIGWAS (miRNA–target gene networks enrichment on GWAS), a pipeline to systematically estimate specific tissue enrichment over the association of miRNA and target gene networks, to analyze genetics of diverse human traits and provide a better understanding of the effects of miRNA on human diseases [8, 9].

The GTEx database contains whole-genome sequences and tissue samples from donors, helping researchers study the relationship between genetic variation and gene expression. The expression quantitative trait loci (eQTL) provided by GTEx explains gene expression variation and provides a better understanding and interpretation of GWAS results. As it is challenging to distinguish reactive and causal expression changes, incorporating eQTL analysis of GWAS data can help identify causal variants that are more prevalent in individuals with a certain trait, as well as find molecular changes within complex traits [10]. It is valuable that cis and trans-eQTLs are identified to find the position of the locus controlling expression of the target gene.

The goal of this study is to analyze patterns of GWAS functional variants located in 3’UTRs across 31 traits selected from our previous study [11] about their relevance in the context over the network between hosting genes and targeting miRNAs, and elucidate the association between the genes harboring these variants and genetic traits. Due to the large amounts of GWAS variants, it is often difficult to process and analyze the data efficiently by hand. In our study, we have created a bioinformatics pipeline that annotates GWAS files automatically by quantifying the number of variants in different categories for any given trait and identifying and characterizing common variants across multiple different traits.

## Materials and methods

ANNOVAR, a software tool, is used to efficiently annotate genetic variants by examining their functional importance on genes and different variant regions [12]. By using gene-miRNA target pairs for 31 traits from MIGWAS, in which miRNA-gene target pairs were identified in our other study [11], we annotated variation for every trait using ANNOVAR to report functional importance of the targeted genes. We categorized the variants and checked for the prevalence of the 3'UTR variant category for each trait. Specifically, genetic variants located in the 3’UTR region could alter the interactions of the UTRs and the miRNAs. To normalize the proportion, we divided the occurrences of 3’UTR by the unique genes as a measurement matrix for comparison.

By utilizing the annotation tool DAVID, we conducted a Gene Ontology analysis on 15 targeted genes harboring 3’UTR variants for Child Obesity traits, which we found had the highest 3’UTR prevalence among the 31 traits. DAVID is a web-accessible program that utilizes a multitude of tools that enables users to translate genome-scale datasets by converting the data into biological meaning [13, 14].

In addition, in order to have deeper insights about GWAS data, we applied eQTL analysis to GWAS Child Obesity results. All tested gene-SNP pairs and significant eQTL pairs in the GTEx cohort (version 8) were downloaded from the GTEx portal. The GTEx dataset includes 581 Adipose Subcutaneous samples and 469 Adipose Visceral Omentum samples with available genotype and gene expression data. The GTEx eQTL mapping results were filtered Storey Q-value < 0.05 and SNPs with minor allele frequency (MAF) > 0.05 in each tissue respectively. A total of 12,795 eGenes in the GTEx Adipose Subcutaneous samples and 10,410 eGenes in the GTEx Adipose Visceral Omentum samples were retained with this filtering.

We also performed GO and KEGG pathway enrichment analyses to investigate the biological function among the 16 miRNA target genes (*ZNF566, RC3H1, KCTD15, FTO, ARSJ, GMEB1, CEP120, PIGN, ETV7, ZBTB40, ETV7, NFIX, WDR55, C1orf173, FAM114A1, ARSJ, CD59, CLVS1*) identified by MIGWAS for the Child Obesity trait [11]. We employed the g:GOSt tool (https://biit.cs.ut.ee/gprofiler/gost) available on the web-based software g:Profiler [15] and considered all annotated genes as background. We used the default option g:SCS method in g:Profiler for multiple testing correction in our analysis.

We then extracted 3’UTR sequences and locations for all genes in the traits from Ensembl Biomar and searched for motifs of each gene using MEME (Multiple EM for Motif Elicitation), a popular tool used to identify signals, or motifs, in genetic DNA/proteins [16]. We compared the motif region to the variant positions identified and reported from ANNOVAR, which elucidates the functional significance of miRNA binding. Variants located in the motif regions of genes likely play a functionally important role in miRNA binding. The matched motifs regions are reported and visualized through various formats like position.

**Fig. 1:**
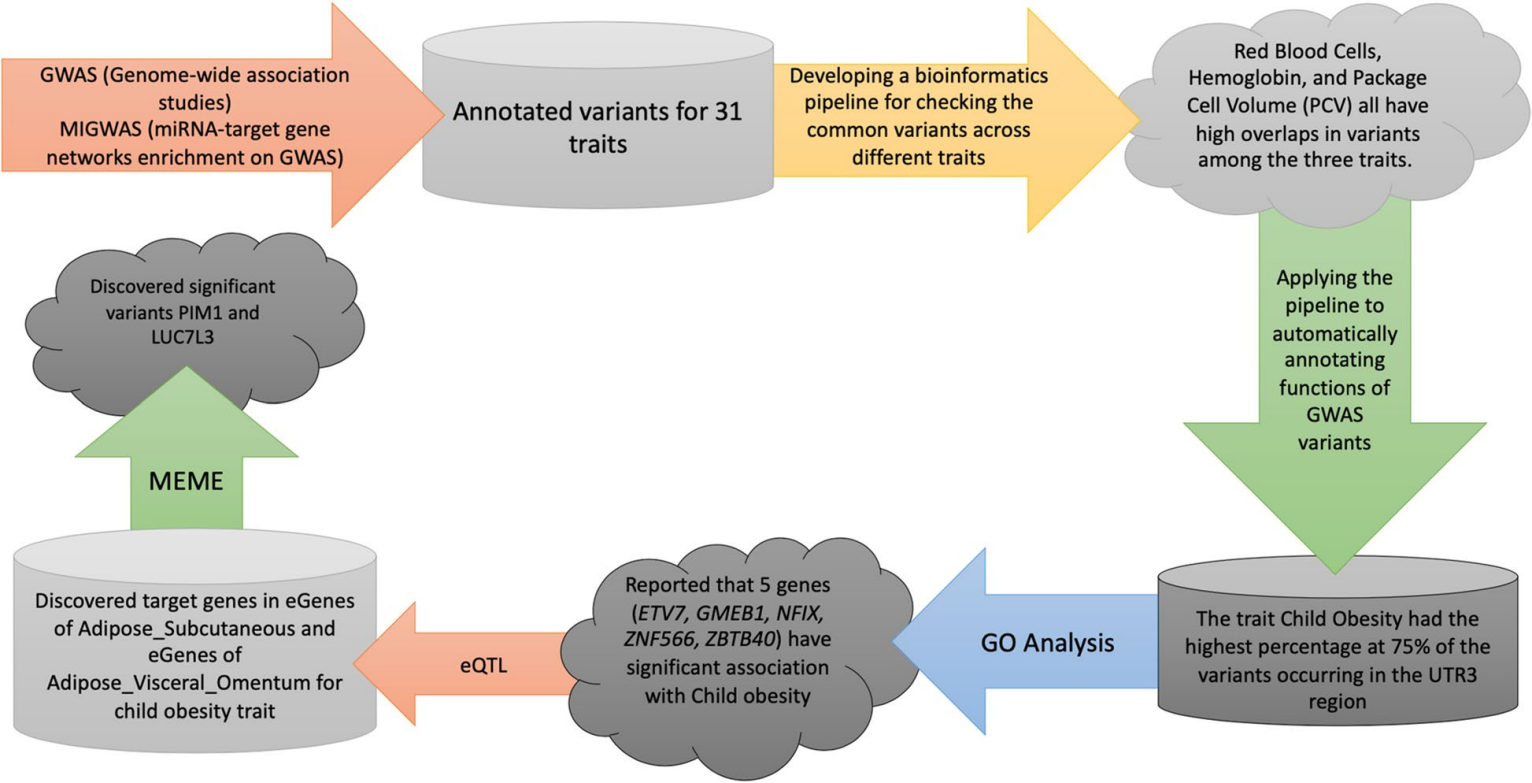
Workflow of elucidating variants in 3’UTR region

The detailed workflow of identifying variants in 3’UTR regions of miRNA-targeted genes for 31 traits is shown in Fig. 1.

## Results

### GWAS variants annotation pipeline (TACG-Var)

**Fig. 2:**
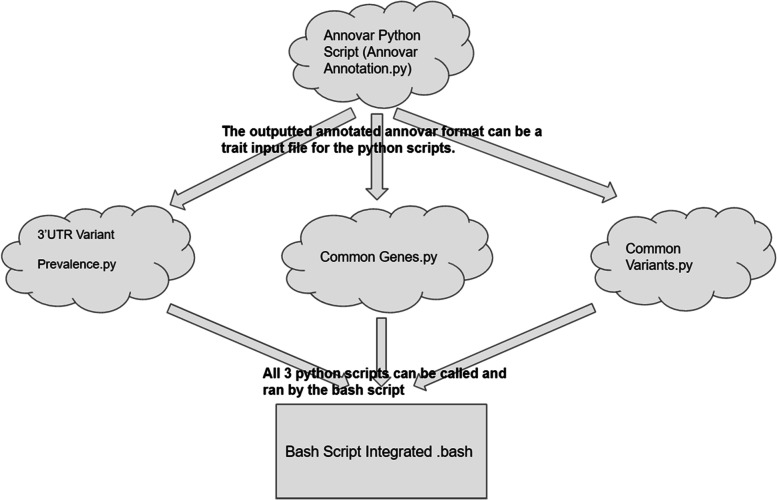
Pipeline calling process of annotating variants for the large number of GWAS traits

Our developed bioinformatics pipeline named as TACG-Var: Tool for Annotation and Classification of Genes and Variants efficiently annotates large numbers of variants files and is available at https://github.com/JuDong1214/Pipeline/blob/main/README.md. The pipeline includes 4 python scripts and 1 bash script, which calls 3 of the python scripts: 3'UTR Variant Prevalence.py, Common Genes.py, and Common Variants.py. The detailed pipeline calling process is shown in Fig. 2.

The ANNOVAR python calling script annotates traits and reports functional importance in ANNOVAR format. The other 3 python scripts, 3'UTR Variant Prevalence.py, Common Genes.py, and Common Variants.py. can be run by the bash script under the same directory. Under a Unix system, one can run the python calling bash script to efficiently run all python scripts on all files under the same directory.

The 3'UTR Variant Prevalence.py script finds the prevalence of 3’UTR regions in each trait. This script takes in ANNOVAR formatted.csv files and counts the number of unique regions (intergenic, intronic, ncRNA_intronic, UTR3, exonic, downstream, upstream, ncRNA_exonic, UTR5, upstream;downstream, splicing, ncRNA_splicing, and UTR5;UTR3) in each ANNOVAR formatted trait file. By utilizing the bash script to automate this process for all traits, the outputs are stored in a created output file.

The Common Variants.py script is used to find the common variants between two individual traits. The bash script runs this script multiple times and automatically compares all the variant files under the same directory. This python script takes in ANNOVAR formatted.csv or.xlsx files, and outputs in standard output format when run alone.

The Common Genes.py script is used to find the common genes between two individual traits. Similar to the process of the common variants script, the bash script runs this script multiple times and automatically compares all the variant files under the same directory. This python script takes in.txt files with a gene name in each line, and outputs in standard output format when running this script alone.

To top off the pipeline, the bash script is needed to automate the 3'UTR Variant Prevalence.py, Common Genes.py, and Common Variants.py script for all the traits. By utilizing this bash script, all outputs from the 4 python scripts can be stored in created output files.

### Overlap analysis of MIGWAS traits

**Fig. 3:**
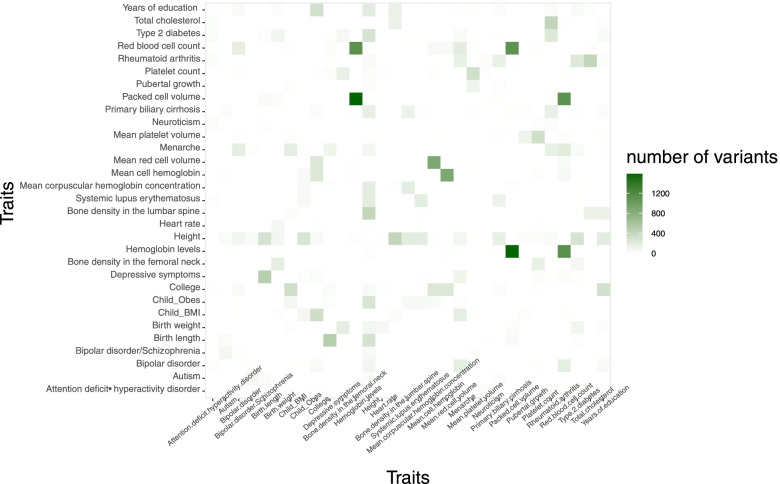
Number of common variants shared between 31 studied traits

Since some variants can be shared across multiple traits, we used the miRNA targeted gene list from multiple traits to check overlap across traits. We found that Red Blood Cells (RBC), Hemoglobin (HB), and Package Cell Volume (PCV) all have high overlaps in variants for miRNA targeted genes among the three traits (shown in Fig. 3). In addition, we found that the Height trait had overlaps with 22 out of the 30 traits. The common variants between 31 MIGWAS traits based on ANNOVAR annotation results are reported in Additional file 1.

Our study found that these three traits have four common genes: *TFPI, HBS1L, MED1, PIK3R3*. *TFPI* encodes a Kunitz-type serine protease inhibitor that initiates the extrinsic pathway of blood coagulation [17]. The intergenic region of *HBS1L* and the MYB gene control fetal hemoglobin level. Additionally, this region influences erythrocyte, platelet, erythrocyte volume and hemoglobin content [18].

### Prevalence of 3’UTR variants and functional enrichment and eQTL analysis on genes associated with child obesity

**Fig. 4:**
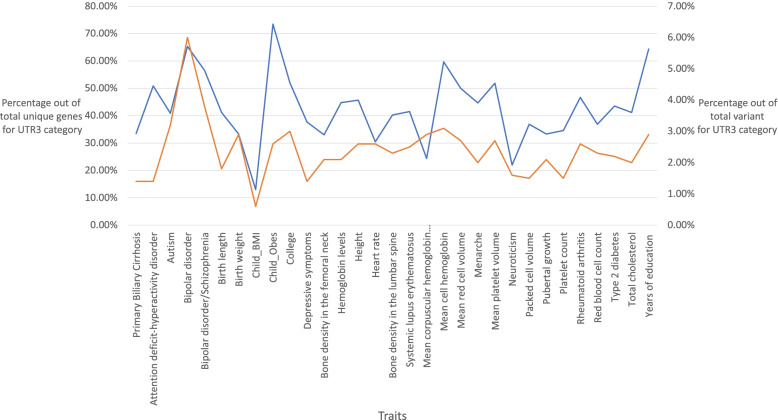
Percentage of genes and variants occurring in the 3'UTR region for 31 traits

Comparing the prevalence of 3’UTR variants in each trait, we identified that Child Obesity had the highest percentage of variants (75%) out of total unique genes occurring in the 3’UTR region (shown in Fig. 4). The detailed number of gene and variants in 3’UTR for 31 MIGWAS traits based on the ANNOVAR results are reported in Additional file 2.

The DAVID analysis discovered that genes *ETV7, GMEB1, NFIX, ZNF566, ZBTB40* with term DNA-binding have significant association with Child Obesity (*p*-value < 0.05).

The result of the list of miRNA targeted genes for Child Obesity trait with significant associated GO term identified as the top cluster by DAVID is shown in Table 1. The detailed DAVID results are reported in Additional file 3.

**Table 1:** Genes and GO terms in the top cluster with a *p*-value less than 0.05 identified by DAVID

Genes	Term	Category	Fold Enrichment	*P*-value
*ETV7, GMEB1, NFIX, ZNF566, ZBTB40*	DNA-binding	UP_KEYWORDS	3.346504065	0.044

We also used eQTL to find the overlap between target genes with eGenes in GTEx v8 eQTL for Child Obesity trait. We discovered that there are 10 target genes in eGenes of Adipose_Subcutaneous and 9 target genes in eGenes of Adipose_Visceral_Omentum. Adipose is a widely studied tissue in child obesity [19, 20]. The overlapping eGenes for two tissues are shown in Table 2. The detailed eQTL results are reported in Additional file 4.

**Table 2:** Targeted genes for tissues Adipose Subcutaneous and Adipose Visceral Omentum

eGenes	Adipose_Subcutanous	Adipose_Visceral_Omentum
*ZNF566*	yes	yes
*KCTD15*	yes	yes
*FTO*	yes	yes
*CEP120*	yes	yes
*PIGN*	yes	yes
*ETV7*	yes	yes
*WDR55*	yes	yes
*FAM114A1*	yes	yes
*CD59*	yes	yes
*CLVS1*	yes	no

We also conducted the functional enrichment analyses using the g:GOSt tool to identify GO and KEGG pathways among the 16 miRNA targeted genes reported for Child Obesity trait. We identified a significantly enriched GO Term (GO:0,008,484 sulfuric ester hydrolaseactivity) with an adjusted *p*-value < 0.05. This GO:0,008,484 sulfuric ester hydrolase activity term is enriched in brown adipose tissue (BAT) [21], and two genes (*ARSJ* and *PIGN*) associated with this term are found to be in the set of intersections. Significant terms and associated genes related to human phenotype ontology (HP) with adjusted p-values were identified and reported in Additional file 5. Interestingly, *PIGN, FTO, and CEP120* associated with HP were also identified through the eQTL analysis as eGenes for Adipose Subcutaneous and Adipose Visceral Omentum tissues.

### Variants in motif region for traits HB and RBC

**Fig. 5:**
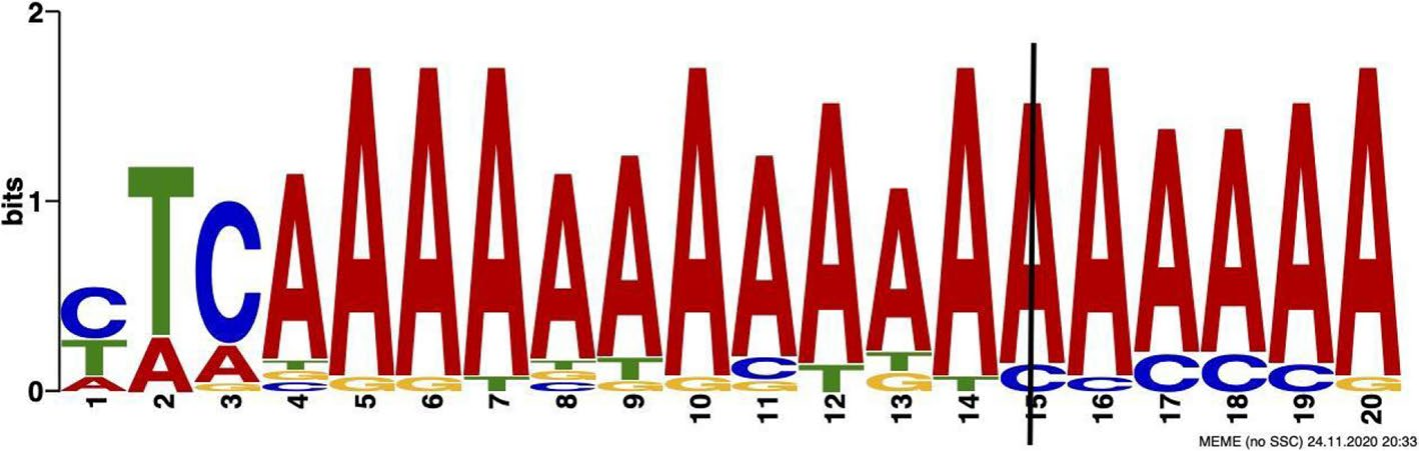
A variant of *PIM1* annotated by ANNOVAR falls within the motif region identified by MEME for Hemoglobin levels (HB) trait

**Fig. 6:**
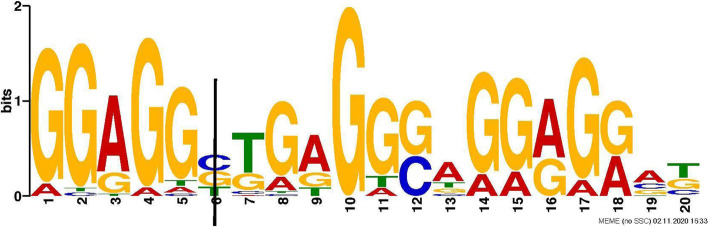
A variant of *LUC7L3* annotated by ANNOVAR falls within the motif region identified by MEME for Red Blood Cell count (RBC) trait

After matching the motifs regions from MEME with the variants positions from ANNOVAR, we discovered that the *PIM1* variant (C > T) occurred at location 37,174,646 inside the HB Motif region 37,174,641–37,174,660 (Fig. 5), and *LUC7L3* variant (T > C) occurred at location 50,753,923 inside RBC Motif region 50,753,918–50,753,937 (Fig. 6). *PIM1* is a threonine kinase often overexpressed in gastric cancers [22], potentially causing HB to be lower than usual. *LUC7L3* is a gene associated with Type 1 Diabetes and codes for a protein that localizes with a speckled pattern in the nucleus [23]. The variant locations inside the Motif region are reported in Additional files 6 and 7.

## Discussions

In our study, we use several bioinformatics tools (MIGWAS, ANNOVAR, MEME, DAVID, eQTL) to elucidate and pinpoint functional variants located in 3’UTRs across 31 traits from GWAS. MIGWAS reported miRNA enrichment over the target gene network for the 31 traits. ANNOVAR annotated the variants harbored by genes associated with the traits and reported the number of variants in the 3’UTR category. MEME provided the motifs regions of the targeted genes, which we used to compare to the variants locations. DAVID was used to conduct pathway analysis on the Child Obesity Trait. eQTL was applied to find tissues relevant to Child Obesity Trait.

In our study, we discovered that Child Obesity had the highest prevalence of variants inside the 3'UTR region. Obesity is strongly tied to distribution of adipose tissue, which mainly accumulates in the intra-abdominal site, which is visceral fat surrounding the omentum, and subcutaneous site [19]. Obesity can also be interpreted as a low-grade inflammatory state with adipose tissue generating large quantities of pro-inflammatory molecules, thus underlying a relationship between the immune system and adipose tissue. For children, obesity is the most common cause of abnormal growth. In addition, children with obesity have higher mortality rates. People who experienced Child Obesity were more susceptible to coronary disease and atherosclerosis.

MiRNAs can regulate gene expression through targeting 3’UTR regions of mRNAs. There were 14 miRNAs (*hsa-mir-320c-2, hsa-mir-10b, hsa-mir-4694, hsa-mir-574, hsa-mir-4458, hsa-mir-653, hsa-mir-3682, hsa-mir-4284, hsa-mir-4297, hsa-mir-4652, hsa-mir-4517, hsa-mir-4421, hsa-mir-4721, hsa-mir-576*) targeting into 3’UTR regions of candidate genes containing variants identified by MIGWAS for Child Obesity trait. A complete list of targeting miRNAs for all traits reported from MIGWAS can be found in our previously published study [11]. Indeed, it has been reported that miRNA seed region could be a key factor to drive the regulation change [6]. The mutation that occurs at miRNA seed and/or binding region could have a serious cascading effect on miRNA's regulation function on its targeting mRNAs/genes.

The five genes identified by DAVID (*ETV7, GMEB1, NFIX, ZNF566, ZBTB40)* that have significant associations with Child Obesity belong to the term DNA-Binding, which refers to proteins that bind to DNA sequences in order to modify the DNA or regulate gene expression [24]. Among the 10 genes associated with Child Obesity and the two adipose tissues identified by eQTL, we noticed that *ETV7* and *ZNF566* were also reported by DAVID. *ETV7* is a member of the ETS family of transcription factors that is involved in oncogenesis and plays an important role in a variety of cellular processes throughout development and differentiation [25]. The protein encoded by this gene is predominantly expressed in hematopoietic tissues [26]. *GMEB1* is a transcriptional factor that is essential for parvovirus DNA replication and modulates the transactivation of the glucocorticoid receptor [27]. *NFIX* is a member of a family of CCAAT-binding transcription factors that can initiate transcription of both vertebrate and viral genes [28]. Malan et al. (2010) reported that there is a nearly ubiquitous expression of *NFIX* in the central nervous system and the peripheral nervous system. Their study of distal femoral growth plates of 1- to 5-week-old mice and human fetus revealed strong *NFIX* expression in bone and in pre-hypertrophic chondrocytes [29].

Our study has several limitations. Firstly, since our method is based on GWAS results, if GWAS misreports, such as only reporting a single variant of the trait instead of the whole genetic region, our results could be affected. Secondly, our pipeline also has many limitations, especially with regards to the format of input files. The files that take ANNOVAR format input require the headings of the graphs to be exactly written as the original ANNOVAR file. Moreover, the output files produce results in text format, so conversion into excel file will have to be done manually.

## Conclusions

Variants located in 3’UTR can often impact gene regulation by altering the binding affinity of miRNA. Therefore, it is important to annotate the functional important variants and see how they are related to their harboring genes and traits. Our study aimed to pinpoint GWAS functional variants located in 3’UTRs across 31 traits selected from our previous study in order to elucidate the association between the genes harboring these variants and genetic traits. The bioinformatics bash pipeline we developed is a valuable tool to annotate a large number of GWAS result files. Lastly, our analysis expands our knowledge on the disease causativeness annotation and provides clarification of non-coding variants’ effect on genetic traits and paves the way for future human disease studies.

## Supplementary Information

**Additional file 1.** Common variants between 31 MIGWAS traits.

**Additional file 2.** Number of gene and variants in 3’UTR for 31 MIGWAS traits.

**Additional file 3.** Genes and GO terms associated with trait child obesity identified by DAVID.

**Additional file 4.** GTEx eQTL mapping results for GWAS genes.

**Additional file 5.** Functional enrichment results from gProfiler for 16 miRNA targeted genes for Child Obesity trait.

**Additional file 6.** Variants in the Motif Region of HB.

**Additional file 7.** Variants in the Motif Region of RBC.

## Data Availability

The input and output datasets associated with the pipeline are available at: https://github.com/JuDong1214/TACG-Var

## Abbreviations

miRNA: Micro ribonucleic acid
RNA: Ribonucleic acid
mRNA: Messenger ribonucleic acid
DNA: Deoxyribonucleic acid
SNP: Single-nucleotide polymorphism
3′UTR: Three prime untranslated region
GWAS: Genome-wide association study
MIGWAS: MiRNA–target gene networks enrichment on GWAS
ANNOVAR: Annotate Variation
DAVID: Database for annotation, visualization, and integrated discovery
eQTL: Expression Quantitative trait loci
